# Biodegradability of Binder System Waste from Hydroxyl-Terminated Polybutadiene Propellant and Pretreatment for Biodegradation

**DOI:** 10.3390/polym18060706

**Published:** 2026-03-13

**Authors:** Kai Wu, Tao Chai, Fei Hu, Zhengmao Ding, Chao Wang

**Affiliations:** 1 Frontier Cross-Disciplinary Research Center for Explosive Science and Microevidence Technology, Shanxi Police College, Taiyuan 030051, China; 18636718168@163.com; 2 School of Environment and Safety Engineering, North University of China, Taiyuan 030051, China; wk2061061@163.com (T.C.); 19890309@163.com (F.H.); 3 Pen-Tung Sah Institute of Micro-Nano Science and Technology, Xiamen University, Xiamen 361005, China

**Keywords:** HTPB binder system, HTPB-based polyurethane, biodegradability, pretreatment, depolymerization

## Abstract

Large amounts of binder system waste are produced upon the recovery of energetic components in scrapped hydroxyl-terminated polybutadiene (HTPB) propellant. This study investigated the biodegradability of the binder system waste using a microbial enrichment solution as the biodegradation medium. We measured the binder system weight loss and performed Fourier-transform infrared (FT-IR), thermogravimetric (TG), and scanning electron microscopy (SEM) analyses of the binder system after 60 days of biodegradation. The results show the binder system film weight decreased by approximately 43% and stabilized after 50 days. The FT-IR analysis shows a reduction in C=O and C-O bond signals, whereas N-H, C-N, and C=C bond signals remain nearly unchanged. The TG analysis shows that the difference between the DOA weight in the initial film and that of the thermal decomposition was almost equal to the weight loss of the binder system film after biodegradation. The SEM analysis shows irregular pits on the film. The binder system has a certain biodegradability, which is mainly caused by its plasticizer component, i.e., DOA. HTPB-based polyurethane, the other major component, is difficult to degrade by microorganisms. As such, the binder system was pretreated with sodium methoxide-methanol solution as a depolymerization reagent, and the pretreated product yielded higher biodegradability.

## 1. Introduction

Hydroxyl-terminated polybutadiene (HTPB) propellant is widely used in weapons and aerospace engines due to its excellent mechanical and energy properties. A large amount of propellant waste is produced when old weapons and aerospace engines are decommissioned due to development, replacement, and aging effects. Because the discarded HTPB propellant still contains high-quality energetic solid components (e.g., ammonium perchlorate, aluminum powder, octogen), recovery is an important and necessary approach to achieve an effective resource cycle and is also an advocated method for the treatment of HTPB propellant waste [1,2,3]. However, the binder system of HTPB propellant has substantially reduced reuse value as post-recycled waste. Binder system treatment methods have therefore become a hot topic in the field of HTPB propellant research.

The binder system of HTPB propellant is a multi-component elastic material formed by the crosslinking and curing of isocyanate and HTPB after adding various functional additives. It is the skeleton carrier of propellant and accounts for 15–20% of the propellant weight [4]. The main components are HTPB-based polyurethane and plasticizer (~95 wt.%), alongside other trace components including catalysts, anti-aging agents, and coupling agents [5]. Landfills and incineration are presently widely used in the treatment of such waste [6,7]. Some studies have investigated pyrolysis treatment, but these remain in the preliminary stage. For example, only liquid distillate temperature and catalyst selection have been tested [8]. In the current environmentally friendly, energy-saving, and low-cost context, effective treatment offers a potential and feasible way to degrade and transform the binder system using microbial metabolic processes, with the binder system as a carbon source to avoid the shortcomings and hazards of existing treatment methods. To address this issue, we investigated the biodegradability of the HTPB propellant binder system and implemented a depolymerization pretreatment protocol to enhance biodegradability. The innovation of this study lies in the comprehensive analysis of the biodegradation process and efficacy of the HTPB propellant binder system through various technical approaches, thereby elucidating the underlying biodegradation mechanism. Addressing the challenge of the HTPB propellant binder system’s recalcitrance to biodegradation, an attempt was made to employ a sodium methoxide/methanol mixture for chemical pretreatment of the binder system. Following this chemical pretreatment, the biodegradability of the material was significantly enhanced. This research provides some basic data and ideas for future research and even the use of biodegradation technology to treat binder system waste, thereby making the biotransformation and environmentally friendly disposal of highly cross-linked polyurethane waste feasible.

## 2. Materials and Methods

Binder system: According to a formulation of HTPB propellant binder system, the components are type-III HTPB (42.7 wt.%), toluene diisocyanate (2.4 wt.%), dioctyl adipate (DOA, 53.6 wt.%), triphenyl bismuth, N,N-diphenyl-p-phenylenediamine, and phosphine oxide. Binder system films with dimensions of 10 × 10 × 1 mm were prepared as the research objects. All the above materials were chemically pure and from Liming Research and Design Institute of Chemical Industry Co., Ltd., Luoyang, China.

The other chemical agents include analytical-grade pure methanol (Tianjin Yuanli Chemical Industry Co., Ltd., Tianjin, China) and analytical-grade pure sodium methoxide (Shandong Xuchen Chemical Technology Co., Ltd., Zibo, China).

Biodegradation medium: Activated sludge was obtained from Taiyuan Beikong Sewage Treatment Plant, mixed evenly, and 10 mL of it was put into beaker A. Then, 1.5 g of contaminated soil obtained from the area for leather waste storage in Taiyuan Houcun Municipal Refuse Landfill was placed into beaker B containing 10 mL of distilled water. The material was stirred to break up the soil and then filtered with five layers of gauze. The filtrate was added to beaker A and agitated. This mixture was diluted with an inorganic salt medium in a ratio of 1:1 by volume to obtain a microbial enrichment solution (i.e., biodegradation medium).

Main instruments: THZ-98A constant temperature oscillation incubator (Shanghai Yiheng Scientific Instrument Co., Ltd., Shanghai, China); AUY220 electronic analytical balance (Japan Shimadzu Instrument Co., Ltd., Kyoto, Japan); Perkin Elmer Spectrum 100 infrared spectrometer (Perkin Elmer Instrument Co., Ltd., Waltham, MA, USA); SU3500 scanning electron microscope (Hitachi Co., Ltd., Tokyo, Japan).

The binder system films were placed in the biodegradation medium and treated to continuous degradation in a constant-temperature oscillation incubator at 30 °C and 160 rpm for 60 days. The films were removed on the 10th, 20th, 30th, 40th, 50th, and 60th days, fully washed with distilled water, and dried to constant weight. The film weight was recorded from an average of three groups of parallel experiments. After each weighing was completed, the films were placed back in the biodegradation medium. The old biodegradation medium was discarded and replaced on the 20th and 40th days of biodegradation. Distilled water was used to replenish the water that was lost in the biodegradation medium due to volatilization and film adhesion. The weight loss of the film, which equaled its initial weight minus its weight at each weighing, was used to evaluate the biodegradation level. A control group was set up using distilled water as the liquid medium to accurately reflect the biodegradability of the binder system film.

Fourier-transform infrared (FT-IR) spectroscopy and thermogravimetric (TG) and scanning electron microscopy (SEM) analyses were carried out on the film after 60 days of biodegradation. The FT-IR tests were conducted using the attenuated total reflection method with a resolution of 4 cm^−1^ over a scanning range of 600–4000 cm^−1^. The TG tests were conducted in air using a heating rate of 10 °C/min, a temperature range of 30–700 °C, and a film weight of 5 mg. SEM analysis was conducted at approximately 500 times magnification.

The following method was used as a pretreatment to enhance binder system biodegradability. First, 15% sodium methoxide by weight of the films was dissolved in methanol with a ratio to the films of 5/1 by weight to prepare a depolymerization reagent. The films were added to the depolymerizer reagent and reacted under reflux at 80 °C and stirring at 350 rpm for 3.5 h. After the reaction was completed, the insoluble depolymerization product in methanol was extracted from the reaction solution, washed with distilled water, and dried to obtain a yellow-brown, viscous fluid product.

The depolymerization product was analyzed by FT-IR analysis, and its biodegradability was evaluated following the aforementioned methods for measuring film weight loss. Because the depolymerization product was sticky, microorganisms adhered to its surface. The depolymerization product was therefore soaked in sodium hypochlorite solution (0.5 wt.% effective chlorine content) for 30 min prior to weighing to kill and remove the adhered microorganisms, and then washed with distilled water. The weight loss of the binder system without DOA was also tested for biodegradation to compare with that of the depolymerization product.

## 3. Results and Discussion

### 3.1. Binder System Biodegradability

#### 3.1.1. Weight Loss of the Binder System Film

**Figure 1:**
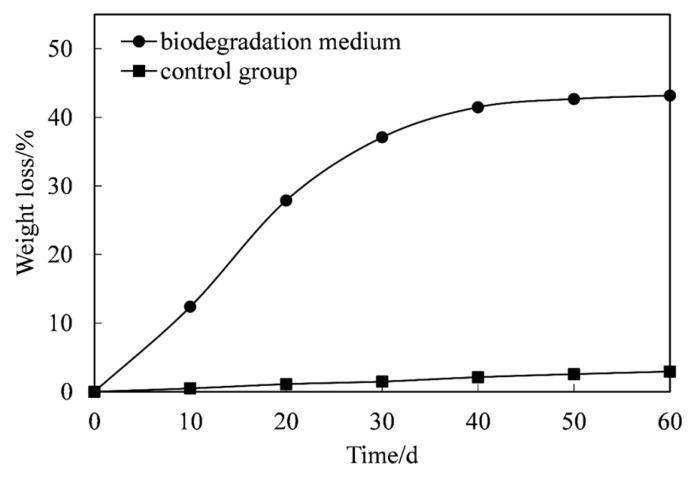
Film weight loss in the biodegradation medium and the control group. Each data point was produced from the average of three parallel experiments.

The weight loss of the binder system film in the biodegradation medium and that of the control group are shown in Figure 1. The weight loss in the biodegradation medium was notable over 60 days. In the first 20 days, the weight loss rate was faster because the microorganisms in the medium adapted to the carbon source of the film and used it for metabolism and reproduction. Between the 20th and 40th days of biodegradation, the weight loss rate slowed and almost entirely stopped on the 50th day, indicating that the film was no longer used and thus no longer being degraded by the microorganisms. The reason might be that only some of the mixed film components degraded owing to the microorganism action, whereas the rest could not be degraded. This also ultimately resulted in an approximately 43% weight loss of the film on the 60th day, compared to the original weight; weight loss did not increase afterwards.

The film weight in the control group was also slightly reduced, which may be attributed to the hydrolysis of certain components within the film matrix under the test conditions. This phenomenon suggests that the film material is affected by abiotic degradation to some extent. However, the weight loss observed for the films exposed to the biodegradation medium was substantially higher than that recorded in the control group. This marked difference clearly indicates the active role of microorganisms in deteriorating the film structure, as microbial activity likely accelerated the breakdown of polymeric components. Consequently, these findings provide strong evidence that the binder system film exhibits a certain degree of biodegradability, supporting its potential for environmental degradation under biologically active conditions.

#### 3.1.2. FT-IR Analysis of the Binder System Film

**Figure 2:**
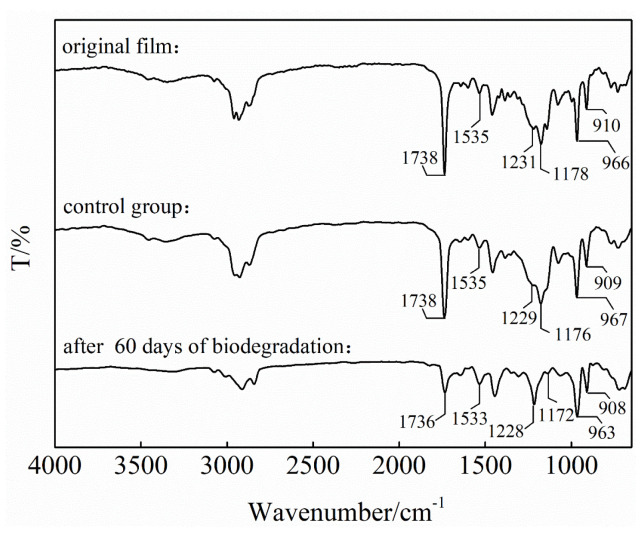
FT-IR spectra of the original binder system film, the film in the control group, and the film after 60 days of biodegradation.

FT-IR spectra of the original binder system film, after 60 days of biodegradation, and those of the control group are shown in Figure 2. In the original film spectrum, the absorption peak at 1738 cm^−1^ is attributed to the stretching vibration of C=O, the one at 1535 cm^−1^ is attributed to the bending vibration of N-H, the one at 1231 cm^−1^ is attributed to the stretching vibration of C-N, the one at 1178 cm^−1^ is attributed to the stretching vibration of C-O-C, and those at 966 and 910 cm^−1^ are attributed to the out-of-plane bending vibration of C-H on the carbon–carbon double bond. The absorption peak at 1738 cm^−1^ is formed by the superposition of C=O absorption from the HTPB-based polyurethane and DOA. The absorption peak at 1178 cm^−1^ is formed by the superposition of C-O-C absorption from the HTPB-based polyurethane and DOA. The absorption peaks at 1535 and 1231 cm^−1^ are the characteristic peaks of the urethane bond. The absorption peaks at 966 and 910 cm^−1^ are the characteristic peaks of the olefin structure in the soft segment of HTPB-based polyurethane.

The position and intensity of each characteristic peak of the control group film did not change significantly compared with the original film, which indicates that its chemical composition remained essentially unchanged with no notable chemical changes in the binder system in distilled water. Weight loss analysis, discussed in Section 3.1.1, indicates that the slight weight loss of the film in the control group was attributed to the hydrolysis of some components in the binder system, which was most likely DOA. However, because hydrolysis only occurred in a localized area on the film surface to a low degree, the differences between the FT-IR spectra of the original and control group films are not significant.

Compared with the original film, the most significant change in the film after 60 days of biodegradation is a reduction in the absorption signals at 1736 and 1172 cm^−1^, which indicates that some C=O and C-O-C bonds were lost and some components in the binder system biodegraded. In contrast, the 1533 and 1228 cm^−1^ absorption signals remained unchanged, indicating that the urethane bonds did not biodegrade. The 963 and 908 cm^−1^ absorption signals did not change, indicating that the olefin structure was also not biodegraded. We therefore consider that the reduction of C=O and C-O-C bonds in the biodegradation is attributed to the breaking of ester bonds in the DOA, whereas HTPB-based polyurethane is difficult to biodegrade. The biodegradability of the binder system is reflected in the DOA component.

#### 3.1.3. TG Analysis of the Binder System Film

**Figure 3:**
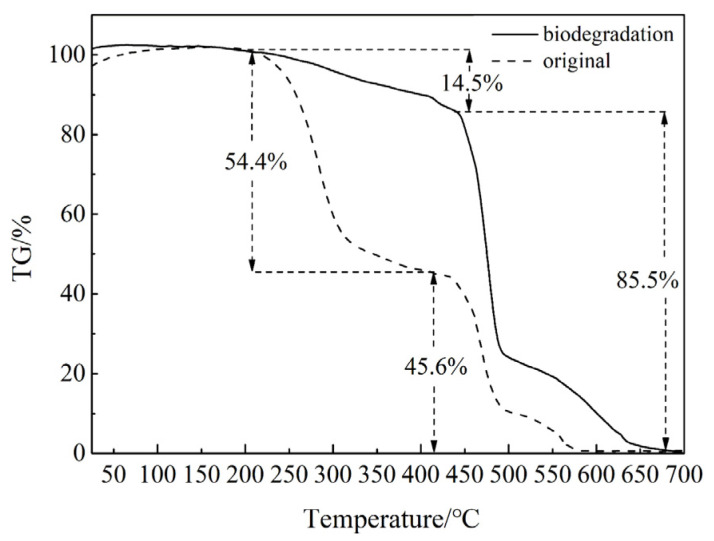
TG curves of the original binder system film and the film after 60 days of biodegradation.

The TG curves of the original binder system film and those after 60 days of biodegradation are shown in Figure 3. There are three weightlessness steps in the original film TG curve. The first weightlessness step occurred at approximately 210 °C due to DOA thermal decomposition with a weight loss of 54.4%, which is nearly consistent with the weight of the DOA in the original film (53.6%). The second and third weightlessness steps were mainly due to the thermal decomposition of the HTPB-based polyurethane, with a total weightlessness of 45.6%. Among them, the second weightlessness step at approximately 414 °C is attributed to thermal decomposition of soft segments of the polyurethane, and the third weightlessness step at approximately 491 °C is attributed to the thermal decomposition of hard segments of the polyurethane. Weight loss between 150 and 400 °C in polyurethane has been attributed to thermal decomposition of hard segments [9], and the onset of thermal decomposition has been reported to occur anywhere between 150 and 260 °C [10]. The soft segments had a higher stability against thermal decomposition than the hard segments [11]. The thermal decomposition characteristics reported in previous studies differ from those obtained here, which may be due to the influence of some factors such as isocyanate and polyol types [12], as well as the atmosphere used during thermal decomposition analysis [13]. The three-dimensional molecular structure of HTPB-based polyurethane may also explain the increase in thermal decomposition temperature.

Three weightlessness steps were also observed in the TG curve of the film after 60 days of biodegradation, and the starting temperatures of weight loss were consistent with those of the original film. The first weightlessness step of 14.5% was caused by the thermal decomposition of DOA. Assuming that “a” represents the initial weight of the original film, after biodegradation, the weight loss of the original film was a × 43%, with the residual weight of a × 57%. The DOA weight of thermal decomposition was therefore approximately 8.3%a (a × 57% × 14.5%). Because the total weight of DOA in the film was 53.6%a, the decomposed DOA in biodegradation was about 45.3%a (53.6%a − 8.3%a), which is nearly consistent with the weight loss (43%) of the binder system film after biodegradation. Combined with the FT-IR analysis, the DOA in the binder system was demonstrated to be the main biodegraded component.

#### 3.1.4. SEM Analysis of the Binder System Film

**Figure 4:**
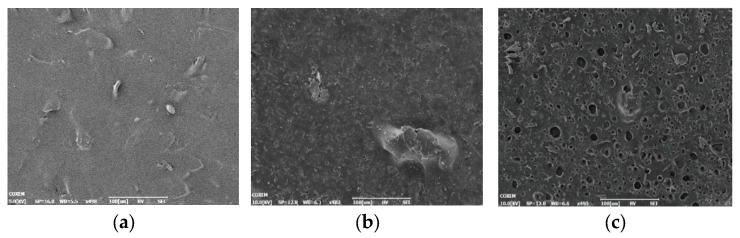
SEM images of the original binder system film, the film in the control group, and the film after 60 days of biodegradation. (**a**): Original binder system film; (**b**): film in the control group; (**c**): film after 60 days of biodegradation.

Film surface topographies of the original binder system, after 60 days of biodegradation, and those of the control group are shown in Figure 4. The surface of the original film was flat and smooth. The surface of the film in the control group was rough with some small bumps and potholes, which might have been caused by the hydrolysis of DOA. The biodegradation signs of the film after 60 days of biodegradation were clearly observed with numerous irregular pits on the film. However, due to the masking effect of the network structure of HTPB-based polyurethane, the closer DOA is inside the film, the more difficult it is for DOA to make contact with the microorganisms or biological enzymes, the latter of which play a role in degradation. Furthermore, it is more difficult to penetrate into the network and combine with DOA, resulting in no biodegradation of the innermost DOA. The use of DOA by the microorganisms was limited, spanning the film surface to a certain depth, and the DOA in this area was almost entirely decomposed. Therefore, on the premise that the HTPB-based polyurethane could not biodegrade, the degradation weight loss stopped on the 50th day, and the film weight tended to be constant thereafter.

### 3.2. Pretreatment of Binder System

#### 3.2.1. Causal Analysis of the HTPB-Based Polyurethane Biodegradation Difficulty

The binder system underwent partial biodegradation, primarily attributed to the small molecular component dioctyl adipate (DOA), which served as a susceptible site for microbial attack. In contrast, the HTPB-based polyurethane within the binder system proved to be significantly more resistant to biodegradation. As a polymeric material, HTPB-based polyurethane exhibits structural characteristics that diminish the capacity of microorganisms to utilize it as a viable carbon source for metabolic processes. This difficulty is further compounded by the molecular architecture of the binder system. During the initial design phase of the propellant formulation, considerable emphasis was placed on achieving optimal mechanical strength, enhanced loading capacity, and long-term storage stability. To meet these performance requirements, the molecular structure of the binder system was intentionally engineered as a three-dimensional crosslinked network [14]. As a result, the HTPB-based polyurethane possesses a high degree of crosslinking and polymerization, which inherently increases its structural rigidity and chemical stability. These same features, however, significantly elevate the difficulty of biodegradation, ultimately rendering the material effectively nonbiodegradable under environmentally relevant conditions.

For the biodegradation of polyurethane, related studies on polyester and polyether polyurethane have shown that they have a certain biodegradability, especially the former, which can be degraded to a certain extent under composting or soil burial conditions, even with a high polymerization degree [15,16,17]. The main structure of degradation is soft segments (polyester) because the ester group has good hydrolysis characteristics and is conducive to the combination of microorganisms and polyurethane [18,19]. The soft segment of HTPB-based polyurethane is polyene, which is a long molecular chain connected by carbon–carbon bonds. Extensive studies have shown that this kind of chemical structure has strong biodegradation resistance, and it is difficult for microorganisms to effectively attack and break the bonds [20,21,22]. At the same time, the existence of numerous carbon–carbon double bonds and the lack of strong electronegative elements in the structure increase the hydrophobicity of polyurethane, which results in an inhibitory effect on microorganisms and enzyme activity. Therefore, the HTPB-based polyurethane demonstrated poor biodegradability, which lends it to being widely used as a biostable polymer material [23].

#### 3.2.2. Principle of Pretreatment

Previous polyurethane biodegradation studies mainly focused on waterborne or linear polyurethane with a low polymerization degree and molecular weight. The urethane bond has been shown to have a certain biodegradability, and the biodegradation of urethane bonds in polyurethane with a linear structure is substantially easier than that with a three-dimensional network structure [24,25]. To improve the biodegradation of HTPB-based polyurethane in the binder system, one potential approach is to reduce the molecular weight and polymerization degree. To this end, the binder system can be depolymerized by chemical methods. With sodium methoxide-methanol solution as the depolymerization reagent, the urethane bonds are broken via the transesterification. The reaction is as follows.

(1) $$\sim N\mathrm{COOC}H_{2}R\sim +CH_{3}\mathrm{OH}\;\overset{\mathrm{Na}\mathrm{OC}H_{3}}{\to }\sim N\mathrm{COOC}H_{3}+\mathrm{HOC}H_{2}R\sim$$

In this process, the crosslinking points in the HTPB-based polyurethane are broken, which forms molecular fragments and meets the requirement for reducing the molecular weight of the substrate as much as possible prior to biodegradation. Moreover, difficult-to-biodegrade urethane bonds are removed, and the inhibition caused by the “bound node” (hard segments of polyurethane) on the movement of the molecular chains is relieved. The depolymerization product, therefore, has a better biodegradability in theory. It should be noted that methyl ester, as a product, was dissolved in methanol, and the hydroxyl-terminated depolymerization product (yellow-brown, viscous fluid product), as a pretreatment product, would biodegrade. In addition, the transesterification of DOA also occurred in this process, generating dimethyl adipate and isooctanol, which were dissolved in methanol.

#### 3.2.3. FT-IR Characterization and Biodegradability of Pretreatment Product

**Figure 5:**
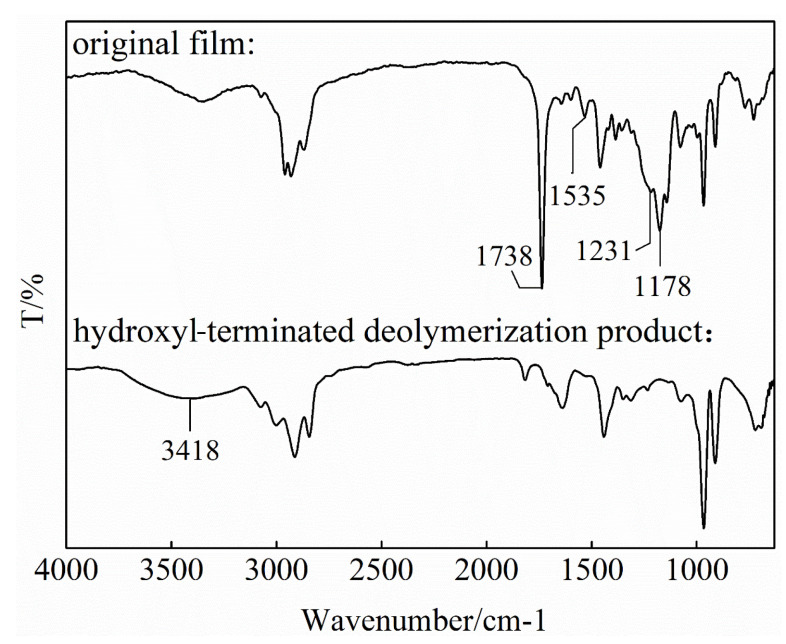
FT-IR spectra of the original binder system film and the pretreatment product.

The FT-IR spectrum of the pretreatment product is shown in Figure 5. Compared with the binder system (i.e., original film in Figure 2), the most notable change in the FT-IR spectrum of the hydroxyl-terminated depolymerization product is that the characteristic C=O (1738 cm^−1^), C-O-C (1178 cm^−1^), N-H (1535 cm^−1^), and C-N (1231 cm^−1^) peaks almost entirely disappeared. This suggests that the urethane bonds in HTPB-based polyurethane and the ester bonds in DOA were successfully broken via depolymerization. In addition, the absorption peak appearing at 3418 cm^−1^ is attributed to OH in the hydroxyl-terminated depolymerization product. Depolymerization strongly reduced the molecular weight and polymerization degree of the binder system, which generated a depolymerization product with a viscous fluid state that is helpful for subsequent biodegradation.

**Figure 6:**
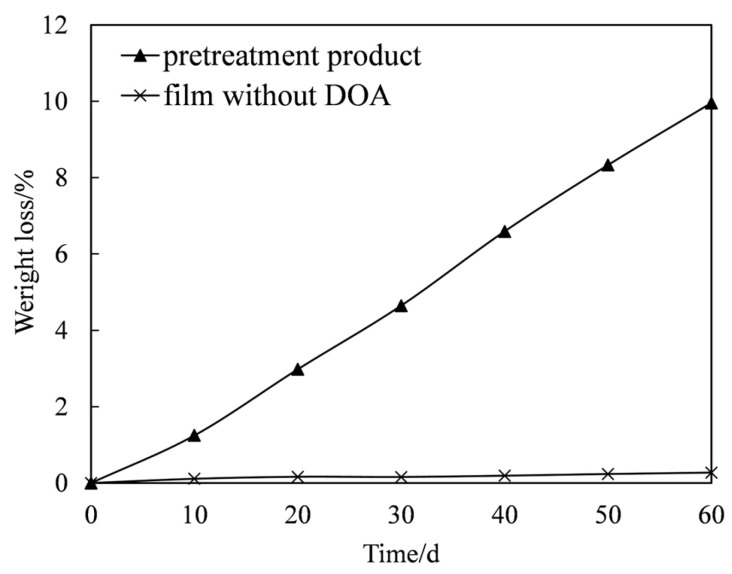
Weight loss of the film without DOA and of the pretreatment product. Each data point was produced from the average of three parallel experiments.

The weight loss of the binder system film without DOA and pretreatment product is shown in Figure 6. As illustrated in the figure, the film formulation without DOA exhibited only minimal weight loss throughout the entire 60-day incubation period, with its weight remaining virtually unchanged in the biodegradation medium. This observation strongly corroborates the earlier conclusion that HTPB-based polyurethane is inherently resistant to microbial degradation, further confirming its recalcitrant nature under the tested conditions. In stark contrast, the pretreated product underwent a substantial weight loss of approximately 10% within the same 60 days. This marked difference clearly demonstrates that the pretreatment process significantly enhanced the biodegradability of the material when compared with the unmodified HTPB-based polyurethane. Nevertheless, despite this improvement, the overall degradation rate remained relatively moderate. This limited rate may be attributed to the fact that the pretreated product, although modified, still fundamentally constitutes a polymeric material characterized by long linear molecular chains. Such structural features likely continue to pose a barrier to rapid microbial assimilation, thereby constraining the extent and speed of biodegradation.

## 4. Conclusions

In this study, the biodegradability of the binder system derived from hydroxyl-terminated polybutadiene propellant waste was systematically investigated. The experimental results demonstrate that the binder system possesses a certain degree of biodegradability, albeit with significant variations in the susceptibility of its individual components to microbial attack. Among the primary constituents, the plasticizer dioctyl adipate was identified as the main contributor to the observed biodegradation, owing to its relatively low molecular weight and accessible ester linkages, which are readily cleaved by microbial enzymes. In contrast, the other major component, HTPB-based polyurethane, exhibited pronounced resistance to biodegradation under the same conditions. This recalcitrance is attributed to its highly crosslinked three-dimensional polymeric structure, which limits microbial accessibility and enzymatic action. Consequently, the findings indicate that microbial treatment alone is insufficient to achieve effective degradation of the complete binder system, highlighting the need for pretreatment strategies to enhance the biodegradability of the HTPB binder system.

To treat the waste binder system via microbial technology and improve its biodegradability, a pretreatment method for HTPB-based polyurethane was also investigated. The experimental results demonstrated that the depolymerization product obtained after pretreatment exhibited notable weight loss during subsequent biodegradation assays, indicating a significant improvement in biodegradability compared to the untreated HTPB-based polyurethane. Although the observed degradation rate was not particularly high under the current experimental conditions, it is anticipated that the biodegradation efficiency of the pretreated product can be substantially enhanced through several targeted approaches. These include the screening and isolation of specific microbial strains with specialized metabolic capabilities; the systematic optimization of degradation conditions, such as temperature, pH, and nutrient availability; and, potentially, the construction of synthetic microbial consortia designed to synergistically break down complex polymeric structures. Given these considerations, future research efforts should prioritize the screening and characterization of microorganisms capable of effectively degrading the pretreated product, as this represents a critical step toward developing a viable bioremediation strategy for HTPB propellant binder system waste.

## Data Availability

The original contributions presented in this study are included in the article. Further inquiries can be directed to the corresponding authors.
